# The population-based oncological health care study OVIS – recruitment of the patients and analysis of the non-participants

**DOI:** 10.1186/1471-2407-8-311

**Published:** 2008-10-27

**Authors:** Ron Pritzkuleit, Annika Waldmann, Heiner Raspe, Alexander Katalinic

**Affiliations:** 1 Institute of Cancer Epidemiology of the University of Luebeck/Germany, Beckergrube 43-47, 23552 Luebeck, Germany; 2 Institute of social medicine of the University of Luebeck/Germany, Beckergrube 43-47, 23552 Luebeck, Germany

## Abstract

**Background:**

The ageing of the population is expected to bring an enormous growth in demand for oncological health care. In order to anticipate and respond to future trends, cancer care needs to be critically evaluated. The present study explores the possibility of conducting representative and population-based research on cancer care on the basis of data drawn from the Cancer Registry.

**Methods:**

A population-based state-wide cohort study (OVIS) has been carried out in Schleswig-Holstein, Germany. All patients with malignant melanoma, breast, or prostate cancer were identified in the Cancer Registry. Epidemiological data were obtained for all the patients and screened for study eligibility. A postal questionnaire requesting information on diagnosis, therapy, QoL and aftercare was sent to eligible patients.

**Results:**

A total of 11,489 persons diagnosed with the cancer types of interest in the period from January 2002 to July 2004 were registered in the Cancer Registry. Of the 5,354 (47%) patients who gave consent for research, 4,285 (80% of consenters) completed the questionnaire. In terms of relevant epidemiological variables, participants with melanoma were not found to be different from non-participants with the same diagnosis. However, participants with breast or prostate cancer were slightly younger and had smaller tumours than patients who did not participate in our study.

**Conclusion:**

Population-based cancer registry data proved to be an invaluable resource for both patient recruitment and non-participant analysis. It can help improve our understanding of the strength and nature of differences between participants and non-respondents. Despite minor differences observed in breast and prostate cancer, the OVIS-sample seems to represent the source population adequately.

## Introduction

Cancer diseases are the second most common cause of death in Germany after cardiovascular diseases, with over 400,000 new cases of cancer diagnosed each year [1]. Rising incidence of cancer is partly due to the ageing population and age-dependency of cancer incidence [2]. At the same time, survival rate continues to improve for most cancer types [3]. The expected increase in the overall prevalence of cancer will soon require substantially more resources for treatment services of cancer patients. To respond to changing demand for oncological procedures, a systematic investigation of cancer care is indicated. Studies from the USA report great differences in the access to and the quality of health care services existing between patient groups, regions, and health care providers [4-13]. Research from Europe suggests differences in tumour stage at the time of diagnosis [14,15], in demand, quality and adequacy of oncological care [16-20].

The OVIS-study (**O**nkologische **V**ersorgung **I**n **S**chleswig-Holstein) is a state-wide research project aimed to examine possible differences in health care in patients with malignant melanoma, breast or prostate cancer from the patient's perspective. Schleswig-Holstein is the northernmost state of Germany with a population of about 2.8 million and only a marginal difference in age structure as compared to the whole of Germany. The study began in November 2002; the recruitment ended in April 2005.

The aims of the study were:

- to collect and present data on medical care (diagnosis, therapy, rehabilitation, aftercare) in patients with malignant neoplasm of breast or prostate and malignant melanoma of the skin who reside in Schleswig-Holstein, Germany,

- to identify differences in medical care with high prognostic relevance, factors associated with them and the extent to which these differences contribute to the outcome,

- to compare reported information on medical care with other data pools and evidence-based reference data (guidelines, indicators of quality).

The reasons for including patients with breast or prostate cancer and malignant melanoma of the skin can be outlined as follows: the present study is intended to cover both sex-specific tumours and a type of cancer which occurs in both sexes. Priority was given to prevalent cancers and tumours of high public interest. Malignant breast and prostate neoplasms are the most frequent tumours among Germans. The incidence of malignant melanoma of the skin was always comparably small until recently. Over the past decades, the incidence of malignant melanoma has been on the rise, increasing the level of professional and public interest in this type of cancer. For the sake of the survey, the selected tumours should be growing slowly, allowing for a time delay of about 18 months between diagnosis and survey contact. To prevent possible biases in recruitment due to incomplete registration [21,22], registration procedure had to be completed by the time of scientific contact.

Research on medical care makes often use of secondary data. However, the definition of the source population appears to be a major problem in studies with secondary data [15,18,23]. The OVIS-study used the population-based Cancer Registry of the state Schleswig-Holstein as a data source for recruitment and comparison to the general population (estimation of representativeness).

The data of cancer patients notified to the Cancer Registry was available on request for scientific purposes. The OVIS study had access to both personal and epidemiological data for all patients. Information was drawn from two data sources in the OVIS-study – the Cancer Registry and the questionnaire consisting of a series of questions on received oncological care.

The present paper gives a short account of the basic data and answers the following questions:

- Is it possible to draw a population-based representative sample by recruiting cancer patients for health care research from cancer registries?

- In which way and to what extent do participants of such studies differ in the most important epidemiological characteristics from the source population?

### Excursus: Healthcare system and cancer registration in Germany

For a better comprehension of this study, it would be appropriate to briefly review the healthcare system, data privacy protection and cancer registration in Germany.

A societal consensus about how **data privacy **should be protected is reflected in the legal system (right to informational self-determination). Storage of personal data and especially linking of personal data from multiple sources requires informed consent. German citizens are not assigned a lifelong social insurance number or a central population registry number that could be used to establish a link to the information they had provided, which renders it difficult to carry out a population-based analysis.

The **German healthcare system **is highly decentralised:

- federal structures (sixteen states having sole responsibility with different legislation)

- widely independent sectors (inpatient, outpatient, specialists in private practice) with self-administration

- separate sectors of reimbursement – statutory (about 250 statutory health insurance companies) and private insurance

There is no systematic exchange of data and no linking between the different sectors, neither on an individual nor on a population-based level. (Full information on treatment is stored in the health insurance data set – accessible to the insured person only – however, person profiles vary significantly from one insurance company to another.)

Although **cancer registration **has a long tradition in some parts of Germany, the mandatory nationwide registration started with the German Cancer Registry Act between 1995 and 1999. All physicians in most states are required to submit a notification to the Cancer Registry of all cancer cases that come to their attention. Cancer registration in Germany is conducted by eleven regional registries regulated by sixteen different cancer registry laws of the federal states. In compliance with data privacy regulations, most registries are divided into two registration offices. The Data Collection Office receives data on cancer patients and is allowed to keep their names, addresses and unique patient numbers, but has to delete medical data. The Data Analysis Office receives anonymous data (no personal data, but a unique number) coupled with medical data from the Data Collection Office. Thus, personal and medical data are kept separately. Linking identifiable individuals to the information they had provided is only possible in case of an informed consent by patient.

## Methods

The OVIS study was designed as a population-based state-wide cohort study of patients diagnosed with cancer in the period between 2001 and 2003 and reported to the Cancer Registry between January 2002 and July 2004, the primary diagnostic codes being C43, C50 and C6 in the ICD-10 classification. The study protocol was approved by the ethics committee of the University of Luebeck (reference no. 01–010). All subjects agreed to participate on a voluntary basis and gave their written informed consent.

### Recruitment

Under state law of Schleswig-Holstein, all instant cancer cases are subject to mandatory notification. Every general practitioner, physician or pathologist in attendance on a person with clinical evidence of cancer is required to report newly diagnosed cancers to the Cancer Registry. The cancer patients about whom they must report have no choice, as the principles of consent does not apply according to law. However, if notification is required, the patient can choose between notification by name and notification by code. In the first case, records of cancer are kept by name in the Cancer Registry. This implies a general consent to be approached to participate in potential research projects. The second choice is coded cancer reporting. Coded identifiers are appended to clinical and personal data (age, sex, place of residence) provided by the patient, making the patient anonymous. All cases reported solely by pathologist belong to this group.

Patients notified by name to the Cancer Registry, who were assumed to have agreed to participate, were contacted via mail. The invitation included a detailed description of the research project, a study questionnaire, a patient's declaration to participate, a declaration form allowing the study researchers to contact the general practitioner (GP) and a pre-addressed stamped return envelope. Non-respondents were sent a maximum of two reminders (week 4 and week 8 from first mailing). If these attempts did not result in a response, vital status or address were ascertained at the Residence Registration Office. Recruitment mailings were done in four waves at intervals of sixth months between 2003 and 2005. Data were requested from the Cancer Registry four times – once for each wave. This was done to prevent major differences in the time span between diagnosis and contact and to achieve efficient data query from the Cancer Registry.

The following are the criteria for inclusion:

- first tumour diagnosis in the period from 2001–01 to 2004–04

- age in the range of 18 to 85 years at diagnosis

- residence in Schleswig-Holstein at diagnosis

- the ICD-10 primary tumour site codes C43, C50 or C61

- patient is female (in case of breast cancer)

**Figure 1:**
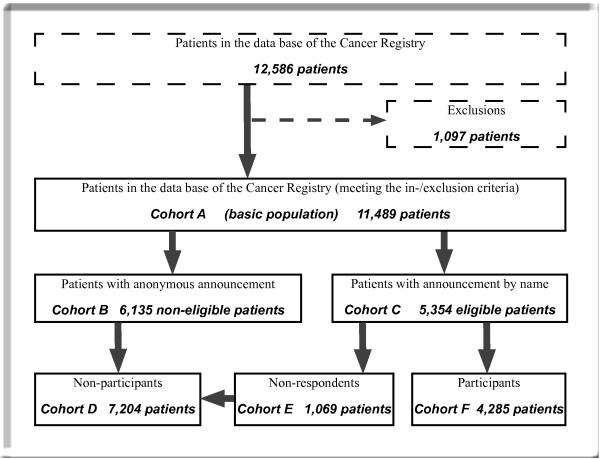
Cohorts of the study.

Exclusion criteria used in selection of research subjects:

Deceased cases were retrospectively excluded from the study and were not part of the source population (cohort A in figure 1). The vital status of non-eligible patients (cohort B in figure 1) was partly unknown. Patients notified by death certificates only were excluded (deceased patients). Subjects may only be enrolled in one cancer site of the study: participants qualified for one cancer site were excluded from participation in other cancer sites.

### Cohorts

Several cohorts have evolved reflecting different ways of notification to the Cancer Registry and willingness to participate (figure 1). Of all patients kept in the Cancer Registry those patients were excluded who do not meet the inclusion criteria. The remaining patients form the source population (cohort A in figure 1). This cohort divides into the cohort of patients who chose notification by code (in the following referred to as non-eligible patients, cohort B in figure 1) and the cohort reported by name (cohort C – referred to as eligible patients). The patients of this latter cohort received the questionnaire. Cohort C was made up of non-respondents (cohort E) and patients who returned the questionnaires (referred to as participants – cohort F). Non-eligible patients (cohort B) and non-respondents (cohort E) form the cohort of non-participants (cohort D) – these were the patients who did not complete the questionnaire. For all cohorts, a relatively small epidemiological data set exists in the Epidemiological Cancer Registry [24]. The data set includes information on diagnosis (ICD-10 coded), histology, tumour stage, age, gender, place of residence, therapies (yes, no), and some other. The above mentioned epidemiological data from the Cancer Registry were used to compare different cohorts.

To assess the representativeness of participants, we compared them to non-participants (cohort D consisting of non-respondents and patients with notification by code) and the source population (cohort A).

### Rates and comparisons

Patients had to decide on study participation twice. At notification to the Cancer Registry patients can give a general consent to be invited to participate in research projects. All patients who refused consent and those notified only by pathologist (pathologists can not ask for consent) cannot decide on their cooperation in the second phase, the second phase being participation in the OVIS study. To account for different decision levels, we introduced two participation rates. The study size is the ratio of participants to the source population in percent (cohort F/cohort A * 100). This ratio can be interpreted as the size of a sample that is activated by recruiting cancer patients from a cancer registry.

The second rate is the ratio of participants to all eligible patients (cohort F/cohort C * 100). This rate can be interpreted as recruitment efficiency [25]. Both rates have to be stratified by sex (in case of C43) and age (all entities). Age was dichotomised by the median age of the source population (cohort A) for each type of cancer – with "young" meaning an age of less than or equal to the median age and "old" meaning an age above the median age.

Decisive with respect to possible bias is the cooperativeness of patients. The existence and (if necessary) the nature of differences between participants (cohort F) and non-participants (cohort D) has been checked. Participants (cohort F) were compared to non-participants (cohort D) to assess the representativeness of the participating cohort (selection bias). A comparison of participants to the source population was not done by reason that the cohort of participants is not independent from the source population. We used only the epidemiological data from the Cancer Registry for comparison because this data was available for all cohorts. Information provided in the questionnaires were not used to improve data stored in the Cancer Registry, such an improvement could generate a bias since data improvement was not possible for cohort D.

We have intentionally refrained from comparison of participants (cohort F) to non-respondents (cohort E) because the number of non-respondents was small compared to the number of non-eligible patients (cohort B), thus shifting away from the focus on representativeness.

### Questionnaire and data handling

Depending on the cancer site, the questionnaire included about 60 questions and requested information on diagnosis, therapy, quality of life, rehabilitation, post-operative treatment, patient's information and socioeconomics. Data obtained from research participants were stored in an Access database. To protect the confidentiality of the information, personal data were separated from study data and password-protected.

Data entry was done by medically trained staff. Range (validity) and logical (plausibility) checks were performed routinely at data entry by the computer program. Any inconsistency or invalid information was verified or corrected manually by data entry staff. Any implausibility within the questionnaires caused by the respondent was corrected by standard procedures (e.g. The questionnaire asked for duration in days and the patient answered in years. We calculated the days.). The quality of data entry was checked by double data entry for a control sample including 5% of all data entries with 179 data fields per single data set. The overall proportion of errors was less than 1%.

### Used scores, categories and quality of life

Social status was determined by using a three level model appropriate for Germany [26]. It is an additive sum score made up of three coded items (highest school leaving certificate, occupational status and household net income). Missing values were substituted by the mean of the two other items. If there was more than one missing value, social status was not computed. The score differentiates between three social levels. It can be considered a simplified form of the frequently used Winkler-Index [27] with comparable results in the distribution.

The spatial aspect was a further factor to be considered in the quality of medical care service. Supply may possibly differ between rural and urban areas. Therefore, comparisons were made between these two categories, which are defined by regional planning state law [28].

The global quality of life/health status was assessed using the EORTC QLQ-C30 [29]. According to the EORTC guidelines, the score was transformed linearly and standardized to a scale ranging from 0 to 100.

### Statistical methods

In the present paper, descriptive statistics (absolute and relative frequencies, mean and median values as well as standard deviations) is used to summarize the data. The Chi-Square- and Mann-Whitney U-tests were conducted to analyse differences between participants (cohort F in figure 1) and non-participants (cohort D).

A multivariate approach was applied to exclude interactions between the variables used in the univariate analysis. The following independent variables were included in a binary logistic regression model: surgery [yes, no, unknown], T-category [T1, T2, T3, T4, unknown], spatial category [rural, urban, unknown], age [years], sex [male, female] in case of C43 and hormone therapy [yes, no, unknown] in case of C61. The outcome variable was the variable participant [yes, no]. All cases that were submitted to the Cancer Registry solely by pathologists (C43: 28 cases, C50: 228 cases, C61: 362 cases) had to be excluded from the multivariate analysis because of missing clinical information.

## Results

In the period from January 2002 to July 2004, a total of 12,586 patients with breast cancer (5,800; 46.1%), prostate cancer (5,185; 41.2%) and malignant melanoma (1,601; 12.7%) were reported to the Cancer Registry of Schleswig-Holstein. 1,097 patients (8.7%) failed to meet the inclusion criteria and had to be excluded from the study. The excluded patients were 98 cases (6.1%) of reported malignant melanoma, 552 cases (9.5%) of breast cancer, and 447 cases (8.6%) of prostate cancer.

The remaining 11,489 patients (91.3% of all notifications) met the inclusion criteria and represent the source population (cohort A in figure 1) of the study. Of those patients who remained in the study, 5,354 patients (46.6%) were eligible for scientific contact (cohort C in table 1).

**Table 1:** Case numbers of the cohorts in the study

	**Melanoma**		**Breast cancer**		**Prostate cancer**	
	***N***	***%***	***N***	***%***	***N***	***%***

Basic population **(cohort A)**	1,503	100	5,248	100	4,738	100
non eligible patients **(cohort B)**	741	49.3	2,882	54.9	2,512	53.0
eligible patients **(cohort C)**	762	50.7	2,366	45.1	2,226	47.0
Participants **(cohort F)**	608	40.5 * [79.8]	1,927	36.7 * [81.4]	1,750	36.9 * [78.6]
Non-respondents **(cohort E)**	154	10.2 [20.2]	439	8.4 [18.6]	476	10.0 [21.4]
Non-participants **(cohort D)**	895	59.5	3,321	63.3	2,988	63.1

* The value without parenthesis describes the study size (Percentage of the participants to the basic population). The value with parenthesis describes the recruiting efficiency proportion (Percentage of the participants to the eligible patients)

The study size – as measured by the ratio of participants (cohort F) to the source population (cohort A) – was 37.3% over all tumour entities based on 4,285 completed self-administered questionnaires. Table 1 differentiates the results by the three analyzed tumour entities. Stratification by sex was only appropriate for malignant melanoma: The study size was 42.9% for men and 38.6% for women (p = 0.093). After stratification by sex and age, the study size was estimated at 38.5% for younger (< = 58 years) and 46.0% for older (59 years and older) men (p = 0.056) and at 39.0% for younger and 38.0% for older women (p = 0.776). In breast cancer patients, the study size amounted to 41.2% for younger women (< = 61 years) and 32.2% for older women (62 years and older) (p < 0.001). The study size was 42.7% for younger men (< = 68 years) and 30.4% for older men (69 years and older) (p < 0.001) in prostate cancer patients.

The recruitment efficiency rate – defined as the ratio of participants (cohort F) to all eligible patients (cohort C) – over all tumour entities reached 80.0%. The recruitment efficiency rate was not different for the sex-specific cohorts. After adjustment for sex and age, the recruitment efficiency rate in melanoma patients was 78.5% for younger and 80.6% for older men (p = 0.644) as well as 82.5% for younger and 76.3% for older women (p = 0.121). The recruitment efficiency rate in breast cancer patients was 85.3% for younger women and 76.5% for older ones (p < 0.001).

In patients with prostate cancer, the recruitment efficiency rate for younger men was 83.3% and 71.6% for older patients, respectively (p < 0.001).

### Characteristics of 4,285 study participants

Table 2 summarises the characteristics of 4,285 study participants including sociodemographics (age, social status, spatial category) and main aspects of medical care (diagnosis, therapy, rehabilitation, global health status). The median age of patients with malignant melanoma differed between the genders, men being on average 62.5 and women 54.0 years of age (p < 0.001). 48.8% of the population of Schleswig-Holstein live in rural and 51.2% in urban areas. This distribution is also present in melanoma and breast cancer patients. Only participants with prostate cancer were more likely to live in an urban area. The median time from diagnosis to survey contact in the context of the OVIS study was 396 days for C43, 525 days for C50 and 481 days for C61, respectively.

**Table 2:** Characteristics of the participants

	**Melanoma**	**Breast cancer**	**Prostate cancer**
**Variable**	**(N = 608)**	**(N = 1,927)**	**(N = 1,750)**
***Age at first diagnosis [yrs]***			
Mean ± SD	55.5 ± 15.7	58.8 ± 11.3	66.9 ± 7.0
Median	59	60	66
***Sex [%]***			
Male	46.1	--	100
Female	53.9	100	--
***Social level [%]***			
Lower	12.4	15.6	14.0
Middle	65.0	67.9	63.4
Upper	22.6	16.5	22.6
	(N = 589)	(N = 1,851)	(N = 1,718)
***Spatial category [%]***			
Rural	48.4	48.6	56.3
Urban	51.6	51.4	43.7
***Time between initial diagnosis and questioning [%]***			
12 months or less	42.9	18.2	23.6
12 to 24 months	43.4	59.5	58.7
more than 24 months	13.7	22.3	17.7
***Health insurance [%]***			
Private	7.7	4.1	9.8
Statutory	92.3	95.9	90.2
	(N = 534)	(N = 1,743)	(N = 1,456)
***Living with spouse [%]***	79.4	73.5	89.5
	(N = 606)	(N = 1,921)	(N = 1,737)
***Detection of cancer [%]***			
By myself or by my partner	60.1	56.9	9.1
By a medical examination	32.8	39.2	82.3
Other	7.1	3.9	8.6
	(N = 594)	(N = 1,914)	(N = 1,713)
***Surgery [%]***	99.3	98.4	70.4
	(N = 602)	(N = 1,918)	(N = 1,657)
***Attendance to a rehabilitation [%]***	6.4	52.3	45.1
	(N = 598)	(N = 1,871)	(N = 1,647)
***Global health status (QLQ-C30)***			
Mean ± SD	74.1 ± 22.9	65.5 ± 22.4	68.4 ± 20.8
Median	83.3	66.7	66.7
	(N = 593)	(N = 1,866)	(N = 1,704)

### Non-respondents (cohort E)

A total of 1,069 patients who were contacted by mail chose to exclude themselves from the proposed study (cohort E in figure 1). The non-respondents were evenly distributed between the three cancer entities accounting for 20% of all eligible patients (table 1). About a quarter of these patients simply did not respond, another 59% returned the questionnaire uncompleted without specifying reasons for not completing the questionnaire and about 5% did not answer for age or health reasons. For 3.6% of all eligible participants, the valid address could not be obtained.

Reasons for non-participation varied only little between the cancer sites. For prostate cancer, we found with 4% a higher value for the category "The study is too complex"/"not convinced of the sense of the study" (3.2% over all three groups). 3.2% of non-respondents with prostate cancer denied their cancer disease (C50: 0.5%, C43: 1.3%) despite having given a written consent for notification by name to the Cancer Registry. About 1.1% of non-respondents with breast cancer did not want to be reminded of the disease and declined their participation. The same reason was found in one melanoma patient but seemed to be completely irrelevant for all prostate cancer patients.

### Comparison of participants to non-participants

**Figure 2:**
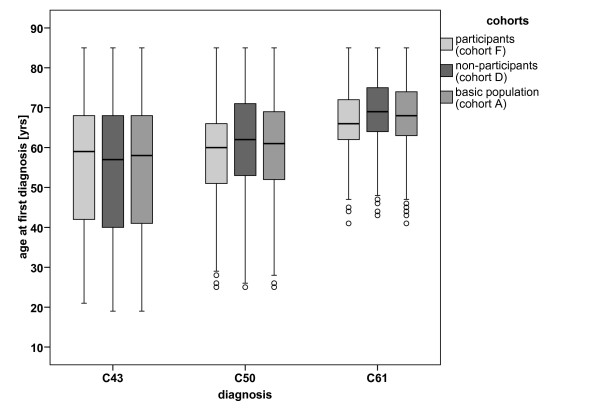
**Age distribution of the study cohorts**. Significance of the difference between participants (cohort F) and non-participants (cohort D): Melanoma: p = 0.341; breast cancer: p < 0.001; prostate cancer: p < 0.001.

Survey participants (cohort F, figure 1) were compared to non-participants (cohort D) to assess the representativeness of the sample. Figure 2 illustrates the distribution of age in the above specified cohorts with respect to the three cancer entities.

After stratification by sex, no significant differences were found in the age distribution of melanoma patients. The difference in the median age between participants and non-participants was a little higher for men (62.5 years vs. 60.0 years; p = 0.141) than for women (54.0 years vs. 53.5 years; p = 0.878).

Table 3 summarises further characteristics of participants (cohort F), non-participants (cohort D), and the source population (cohort A). More women than men were registered with malignant melanoma, but men were more likely to be respondents than non-respondents (46.1% vs. 41.7%).

**Table 3:** Comparison of epidemiological items between the study cohorts/representativeness

**Characteristics**	**Basic population (cohort A)**	**Respondents (cohort F)**	**Non-participants (cohort D)**	**p-value for difference between cohort F & D**
	**%**	**%**	**%**	
***Melanoma***				
N	1,503	608	895	
Sex				0.093
Male	43.4	46.1	41.7	
Female	56.6	53.9	58.3	
Surgery				0.877
Yes	87.9	88.3	87.6	
No	0.4	0.3	0.4	
Unknown	11.7	11.3	12.0	
T-category				0.208
T1	30.9	30.9	30.8	
T2	11.8	10.7	12.5	
T3	8.4	9.0	8.0	
T4	2.7	1.6	3.4	
Unknown	46.2	47.7	45.3	
Spatial category				0.261
Rural	46.6	48.4	45.4	
Urban	53.4	51.6	54.6	
	(N = 1,500)		(N = 892)	
***Breast cancer***				
N	5,248	1,927	3,321	
Surgery				< 0.001
Yes	88.8	93.2	86.3	
No	1.9	0.5	2.8	
Unknown	9.2	6.3	10.9	
T-category				< 0.001
T1	48.4	53.8	45.3	
T2	34.4	34.6	34.3	
T3	4.8	4.5	5.0	
T4	6.9	3.4	9.0	
Unknown	5.4	3.7	6.4	
Spatial category				< 0.001
Rural	52.1	48.6	54.2	
Urban	47.9	51.4	45.8	
	(N = 5,228)		(N = 3,301)	
***Prostate cancer***				
N	4,738	1,750	2,988	
Surgery				< 0.001
Yes	46.3	56.4	40.5	
No	19.8	17.6	21.0	
Unknown	33.9	26.0	38.5	
Hormone therapy			< 0.001	
Yes	31.5	28.2	33.4	
No	28.2	34.3	24.6	
Unknown	40.3	37.4	42.0	
T-category				< 0.001
T1	15.9	15.0	16.4	
T2	39.0	45.8	35.0	
T3	22.8	28.5	19.5	
T4	1.9	1.5	2.2	
Unknown	20.4	9.3	27.0	
Spatial category				0.124
Rural	54.8	56.3	54.0	
Urban	45.2	43.7	46.0	
	(N = 4,718)		(N = 2,968)	

A multivariate analysis was performed for each tumour entity. The results of multivariate analyses were found to be in agreement with the univariate results (table 3) with just one exception – the item "surgery" for prostate cancer was not included in the final model.

### Deceased patients

Patients deceased by the time of survey contact were excluded from the OVIS-study in agreement with the exclusion criteria. The vital status of patients with anonymous notification (cohort B) to the Cancer Registry was unknown, with the consequence that possibly dead could not be excluded from cohort B. In consideration of representativeness issues, some epidemiological characteristics of patients notified by name and deceased by the time of survey (and then excluded) are given in the following. Seventeen patients with malignant melanoma died in the time span between diagnosis and survey contact. Their median age at primary diagnosis was 73 years. The 116 deceased patients with breast cancer (median age 63.5 years) showed the following distribution of the T-category: T1 = 22.4%, T2 = 35.3%, T3 = 13.8%, T4 = 19.8% and unknown = 8.6%. The proportion of operated patients was 81.9%. Deceased prostate cancer patients (n = 109) had a median age of 72 years (T1 = 20.2%, T2 = 30.3%, T3 = 15.6%, T4 = 8.3% and unknown = 25.7%), 24.8% were operated (not operated = 32.1%, unknown = 43.1%). A hormone therapy was received by 62.4% (no hormone therapy = 12.8%, unknown = 24.8%).

## Discussion

The aim of the OVIS-study is the presentation and evaluation of the medical care situation of cancer patients diagnosed with malignant melanoma, breast and prostate cancer. In this paper, the data was critically analyzed in terms of representativeness. Possible biases (e.g. selection bias) were identified and have to be considered when evaluating the results of further analyses.

### Study approach

The objective of the OVIS-study is to survey all patients with the neoplasms in question residing in the study area. The population-based approach gives a better guarantee that the data is representative and that no systematic exclusions of whole patient groups take place. About 5,000 patients participated in the OVIS-study, thus resulting in adequate numbers for subgroup analysis. Subgroup analyses were essential for description and analysis of medical care and provided answers to questions like: "Does the place of residence determine how medical care is delivered?" and "Do patients utilize medical care differently and what is the outcome?"

Records of cancer from the Epidemiological Cancer Registry were used as a data source for patient recruitment. Two reasons were decisive in this choice. Firstly, the Cancer Registry is the most complete data source for population-based research. More than 95% of all expected cases of the chosen cancer sites are registered. All other possible data sources (e.g. clinical cancer registries, doctors or unsystematic questionings) are less complete, suggesting a stronger selection bias. Secondly, the already existing epidemiological dataset was available. This dataset is existent not only for patients reported by name to the Cancer Registry but for all patients included in the Registry, which allows comparison between participants and non-participants as well as between participants and the source population. Whereby not only can the representativeness of the sample be estimated, but possible biases can be detected and described in terms of strength and direction.

One of the arguments against the use of cancer registry data for patient recruitment is a time lag of 12 to 18 month between first diagnosis and contact: The delay is due to untimely notification and high processing time (data entry, enquiries, record linkage etc.) inside the Cancer Registry. The counter-argument is that other preponderate problems (e.g. incomplete registration, missing reference population data) might exist, even if other data sources did not have this problem. Two effects result from this time-lag. On the one hand, a survival cohort is questioned – given that the cancer sites of interest have a high relative survival rate (> 90% one-year-survival, [30]), that bias should be small. On the other hand, recall-bias might occur due to the time-lag. However, cancer is thought to be a very traumatic experience that it can be assumed that patients remember diagnosis, therapy and aftercare very well. Few comparative data exist to corroborate this assumption [31-33]. For a small subsample (56 cases) of the OVIS-study, the same data were collected during the hospital stay and again 12 month later, which allowed validity analysis. The analysis showed an excellent concordance between information given during initial hospital stay and 12 months after diagnosis [34]. A smaller time-lag, attributable to early notification by dermatologists in the study region, was observed for malignant melanoma.

### Representativeness

High response rate is a major determinant of representativeness. The recruitment efficiency rate of about 80% achieved in the OVIS-study is adequate and comparable to other studies. It happens to be a little higher than in the investigation of breast cancer patients based on SEER-data that was conducted by Janz et al. [35], and higher than Karakiewicz et al. [36] reported for prostate cancer patients from Quebec, Canada. However, it is lower than response rates reported for patients with breast cancer and melanoma by Lehto et al., based on hospital data in Finland [37]. Arndt et al. from Saarland, Germany, found similar response rates for patients with colorectal cancer [38] and breast cancer [39].

The principal question addressed is whether there are differences between participants (cohort F) and non-participants (cohort D). Consideration of representativeness was a major factor motivating this comparison. To evaluate representativeness, univariate and multivariate approaches were employed to analyze the epidemiological variables. Just minor differences were observed in the results of both approaches (see below).

There is another important aspect to consider when evaluating representativeness. The cohort of participants is a pure survivor cohort, while deceased persons are possibly included in the non-eligible group. Vital status information could only be obtained for patients who were reported by name to the Cancer Registry – according to the exclusion criteria deceased patients were excluded from the OVIS-study. Hence, patients of the cohorts E and F were alive at the time of questioning. In case of notification by code to the Cancer Registry, vital status could not be determined with certainty (exceptions are cases notified by death certificate only – the so called death-certificate-only cases). It might be the case that some anonymously notified patients deceased in the period between diagnosis and survey contact. Deceased (and excluded) patients reported by name were older and had a higher tumour stage compared to respondents. The cohort of non-participants (cohort D) is composed of about 15% non-respondents (cohort E, deceased patients excluded) and about 85% non-eligible patients (cohort B, including an unknown number of deceased patients). Under the well-founded assumptions that cohort B included some (possibly the same proportion of) deceased patients and that these might have similar characteristics as the excluded deceased patients (older, higher tumour stage), it is obvious that the detected and shown differences between participants and non-participants might be statistically more preponderant than in (clinical) reality.

Knowing the vital status of all patients would have possibly leaded to minor differences between the cohorts. However, the relevance of this effect is different for the cancer sites in question. It appears to be less pronounced in patients with malignant melanoma for the reason of lower median age and survival probability associated with it. It is most preponderant in prostate cancer patients due to higher age and comorbidities.

### Malignant Melanoma

Participants with malignant melanoma were very similar to the source population, with a difference of 2.5 years in median age, men deviating more strongly from the median than women. It can be assumed that a slightly higher response rate in older men (recruitment efficiency rate: 80.6%) brought about this difference. Why older men were more willing to participate remains unclear, but there might be an influence of the variables "living with spouse" and/or "education". On the whole, the observed differences are so small that participants with malignant melanoma in the OVIS-study seem to be a representative sample of the source population.

### Breast cancer

In patients with breast neoplasm, participants and non-participants differed statistically significantly in every demographic category used to evaluate representativeness. Whether these differences were clinically relevant (leading to an unrepresentative sample) has to be examined. Participants were on average 2 years younger than non-participants, which have to be considered in the interpretation of results. No relevant and systematic influence of age difference on the results of the study could be observed. The variable 'age' is regarded as a confounder in most of the statistical analyses. The same is true for the variable 'tumour stage' (T-category). Here again, the differences are statistically significant. There were nearly no differences found for T2- and T3-category, but participants appeared to have more frequently T1-tumours and less frequently T4-tumours. In further analyses, the T-category will be used as a confounder. In reality, the difference will be less pronounced as expected due to the survival effect (see above). The ratio of deceased (excluded) patients (all notified by name) to all eligible patients (cohort C) was about 5% (116 to 2366+116). Deceased patients were on average 3,5 years older than participants and 1,5 years older than non-respondents and had a considerably higher proportion of T3 and T4 tumours.

As shown in Table 3, nearly all patients had undergone surgery. Due to the study size the observed difference of 7% is statistically significant. This difference can hardly be regarded as clinically relevant as most patients (~90%) were operated. It cannot be, however, ignored in specific analyses. The relevance of the variable 'spatial category' for the representativeness of the sample can not be finally evaluated here. The difference is less pronounced but significant. It could be relevant if substantial regional distinctions were analysed between rural and urban areas. Initiated but not finished analyses do not show such divergences between rural and urban areas. By large, the sample can be regarded as representative with a few limitations that can be controlled (age, tumour stage).

### Prostate cancer

Participants with prostate cancer were on average four years older than non-participants. This is a relatively considerable discrepancy, given that the above described survival effect might have a major impact on this cancer site because of the median age of these patients (the median age in the source population was 68 years). Therefore, the real difference should be less pronounced. The ratio of deceased (excluded) patients (all notified by name) to all eligible patients (cohort C) was nearly 5%. Deceased patients were on average 6 years older than participants and 3 years older than non-respondents. They had a higher proportion of T1 and T4 tumours. After exclusion of deceased non-participants (which was not possible, though), the proportion of T4 tumours in non-participants would have been smaller. There is a considerable difference in the category "unknown tumour stage" between participants and non-participants (3 times higher), which can be ascribed to the chosen way of notification. About 12% of non-participants were notified only by laboratory. In such cases, the tumour stage remains largely unknown. Notification by name only by laboratory is impossible. There is no contact to the patient. Notification by name requires necessarily notification by clinician. If only cases with known tumour stage were analyzed, under the assumption that unknown cases were not biased, the differences in the tumour stage distribution between participants and non-participants would be less pronounced. Similarly, the variables "surgery" and "hormone therapy" differed between both cohorts. It seems that operated patients were more willing to participate in the study than patients treated with a hormone therapy. Simply said, patients treated curatively tend to be operated more often and patients treated palliatively were likely to receive a hormone therapy – both are correlated with age and tumour stage. The conclusion to be drawn, therefore, is that the kind of treatment has to be considered in statistical analyses. On the whole, the sample reached a high degree of representativeness for the source population.

The assessment of representativeness in terms of socio-economic criteria remains incomplete since these variables are not part of the Cancer Registry. One of the objectives of the OVIS-study is to determine whether variables like "living with spouse", "social level", "kind of health insurance" have any effect on medical care in patients with malignant neoplasms.

## Conclusion and prospects

The OVIS-study is one of the largest population-based studies focussing on the situation of medical care of cancer patients in Germany. The Epidemiological Cancer Registry as a data source for recruiting proved to be useful to estimate the extent and nature of bias effects. It could be shown that there were just minor differences between participants and non-participants in terms of age and tumour stage distribution. Hence, both factors allow to be controlled for in further multivariate analyses. It is intended to perform a follow up survey in two years.

## Competing interests

The authors declare that they have no competing interests.

## Authors' contributions

RP; Has been made substantial contributions to acquisition of data, analysis and interpretation of data; Has been involved in drafting the manuscript; Has been given the final approval of the version to be published. AW; Has been made substantial contributions to acquisition of data, analysis and interpretation of data; Has been involved in drafting the manuscript. HR: Has been made substantial contributions to conception and design; Has been involved in revising the manuscript critically for important intellectual content.

AK; Has been made substantial contributions to conception and design; Has been made substantial contributions to acquisition of data, analysis and interpretation of data; Has been involved in revising the manuscript critically for important intellectual content.

## Pre-publication history

The pre-publication history for this paper can be accessed here:


